# A novel in vitro *Caenorhabditis elegans* transcription system

**DOI:** 10.1186/s12860-020-00332-8

**Published:** 2020-11-30

**Authors:** Phillip Wibisono, Yiyong Liu, Jingru Sun

**Affiliations:** 1 Department of Biomedical Sciences, Elson S. Floyd College of Medicine, Washington State University, Spokane, WA USA; 2 Genomics Core, Washington State University, Spokane, WA USA

**Keywords:** In vitro transcription, *Caenorhabditis elegans*, Balch homogenizer, Subcellular fractionation, Non-radioactive detection

## Abstract

**Background:**

*Caenorhabditis elegans* is an excellent model organism for biological research, but its contributions to biochemical elucidation of eukaryotic transcription mechanisms have been limited. One of the biggest obstacles for *C. elegans* biochemical studies is the high difficulty of obtaining functionally active nuclear extract due to its thick surrounding cuticle. A *C. elegans* in vitro transcription system was once developed by Lichtsteiner and Tjian in the 1990s, but it has not become widely used, most likely because the transcription reactions were re-constituted with nuclear extract from embryos, not from larval or adult worms, and the method of Dounce homogenization used to prepare the nuclear extract could lead to protein instability. Besides Dounce homogenization, several other techniques were developed to break worms, but no transcription reactions were re-constituted following worm disruption using these approaches. A *C. elegans* transcription system with effective preparation of functionally active nuclear extract from larval or adult worms has yet to be established. Additionally, non-radioactive methods for detecting transcription as alternatives to the conventional radioactive detection also need to be adapted into such an in vitro system.

**Results:**

By employing Balch homogenization, we achieved effective disruption of larval and adult worms and obtained functionally active nuclear extract through subcellular fractionation. In vitro transcription reactions were successfully re-constituted using such nuclear extract. Furthermore, a PCR-based non-radioactive detection method was adapted into our system to either qualitatively or quantitatively detect transcription. Using this system to assess how pathogen infection affects *C. elegans* transcription revealed that *Pseudomonas aeruginosa* infection changes transcription activity in a promoter- or gene-specific manner.

**Conclusions:**

In this study, we developed an in vitro *C. elegans* transcription system that re-constitutes transcription reactions with nuclear extract of larval or adult worms and can both qualitatively and quantitatively detect transcription activity using non-radioactive approaches. This in vitro system is useful for biochemically studying *C. elegans* transcription mechanisms and gene expression regulation. The effective preparation of functionally active nuclear extract in our system fills a technical gap in biochemical studies of *C. elegans* and will expand the usefulness of this model organism in addressing many biological questions beyond transcription.

**Supplementary Information:**

The online version contains supplementary material available at 10.1186/s12860-020-00332-8.

## Background

*Caenorhabditis elegans* is a free-living, 1-mm-long, nematode worm found in soil and decaying organic matter. In 1963, Sydney Brenner proposed research into *C. elegans*, stating that “I would like to tame a small metazoan organism to study development directly” [1]. Since then, *C. elegans* has been used as a model organism to address a wide range of biological questions such as those relating to development, metabolism, neurobiology, and aging. The nematode has many characteristics that make it an excellent model system, including, but not limited to, its rapid (3-day) life cycle, small size, ease of laboratory cultivation, genetic tractability, invariant lineage, effectiveness of RNA interference, and transparent body that allows for monitoring development or gene expression with single-cell resolution. Research involving the use of *C. elegans*, including Brenner’s work on organ development, was awarded with the Nobel Prize in 2002, 2006, and 2008.

Because of its genomic simplicity and physical characteristics, *C. elegans* offers a unique system to study transcription mechanisms and regulation. For example, mutations in pre-initiation complex genes were recovered in genetic screens of *C. elegans* and linked regulation that involves these factors to specific biological processes [2]. The nematode is transparent throughout its entire life cycle making it an ideal system to use fluorescent protein reporters to monitor gene expression in live animals [3]. Despite these advantages, however, the nematode’s contributions to biochemical elucidation of eukaryotic transcription mechanisms have been limited, whereas other model systems, such as *Saccharomyces cerevisiae*, *Drosophila melanogaster*, and cultured mammalian cells, were among the major contributors [2]. In fact, very few biochemical studies of *C. elegans* transcription have been performed. One of the biggest obstacles for such studies was the high difficulty of obtaining functionally active nuclear extract due to the nematode’s thick surrounding cuticle. Therefore, most analyses of transcription mechanisms in *C. elegans* employed intact embryos or whole animals [2]. The nematode cuticle is an exoskeleton composed of predominantly cross-linked collagens, cuticlins, glycoproteins, and lipids [4]. It is synthesized five times during *C. elegans* life cycle: during late embryogenesis prior to hatching and at the end of each of the four larval stage prior to molting, making *C. elegans* at all stages resistant to buffer extraction or mechanic forces [4]. A *C. elegans* in vitro transcription system was once developed by Lichtsteiner and Tjian in the 1990s [5, 6], but it has not become widely used, most likely because the transcription reactions were re-constituted with nuclear extract from embryos, not from larval or adult worms, and the method of Dounce homogenization used to prepare the extract could lead to protein instability [7]. Besides Dounce homogenization, several other techniques were also described to break worms, including pressure cycling technology, bead beating, grinding after flash cooling, sonication, and Balch homogenization [7–11]. Most of these methods have their own advantages and disadvantages and there are no reports of transcription reactions being re-constituted following worm disruption using these approaches. A *C. elegans* transcription system with effective preparation of functionally active nuclear extract from larval or adult worms has yet to be established.

Eukaryotic transcription is a complex biochemical process catalyzed by three nuclear RNA polymerases that synthesize different types of RNA: RNA polymerase I (Pol I) catalyzes the transcription of all rRNA genes except 5S; Pol II synthesizes mRNAs and many non-coding RNAs, including small nuclear RNAs; and Pol III makes tRNAs and other small non-coding RNAs, including 5S rRNA and U6 small nuclear RNA [12]. While RNA Pol I and Pol III have been studied very little in *C. elegans*, the Pol II-mediated mRNA transcription appears to be conserved in the nematode [2]. As in other eukaryotes, mRNA transcription in *C. elegans* includes three phases: initiation, elongation, and termination. The first step is assembly of a pre-initiation complex on promoter DNA, followed by DNA opening and synthesis of a short initial RNA oligomer. Pol II then uses the DNA template to extend the growing RNA chain in a processive manner. Finally, DNA and RNA are released during termination and Pol II can then be recycled to re-initiate transcription. These basic mRNA transcription mechanisms are believed to be conserved in *C. elegans* largely because most of the core transcription factors are conserved in *C. elegans* at the DNA sequence level, and because in vivo studies of the nematode’s transcription machinery components generally yielded results consistent with mechanistic functions that were defined by in vitro studies of other systems [2]. Establishing an in vitro *C. elegans* transcription system is necessary to biochemically identify similarities and differences in transcription between *C. elegans* and other eukaryotes, and more importantly, to further elucidate transcription mechanisms and gene expression regulation.

In the current study, we developed an in vitro *C. elegans* transcription system with effective disruption of larval or adult worms and preparation of functionally active nuclear extract. Traditionally, the detection of transcription activity relied on radioactive labeling of the newly synthesized RNA and visualization of incorporated radioactivity by autoradiography [13]. However, radioisotope labels have many drawbacks including perceived health concerns, regulatory requirements, disposal problems, and short shelf life [14]. While technological advancements allowed the use of non-radioactive methods for RNA detection, such as quantitative reverse transcription PCR (qRT-PCR) [15] or the use of fluorescent nucleotides to label newly synthesized RNA [16], these new methods were mainly developed to investigate transcription in mammalian cell models. Here, we adapted a PCR-based non-radioactive method for either qualitatively or quantitatively detecting RNA in our in vitro system. By coupling in vitro transcription reactions using *C. elegans* nuclear extract preparations with these non-radioactive RNA detection approaches, we examined how pathogen infection affects *C. elegans* transcription and revealed that infection with *Pseudomonas aeruginosa* strain PA14, a human opportunistic pathogen, changed the nematode’s transcription activity in a promoter- or gene-specific manner. Overall, we showed that our in vitro system can be useful for biochemical studies of *C. elegans* transcription. Our approach for effective disruption of larval or adult worms and preparation of functionally active nuclear extract could also expand the usefulness of the *C. elegans* model to biochemically address other biological questions.

## Results

### Preparation of *C. elegans* nuclear extract

**Fig. 1:**
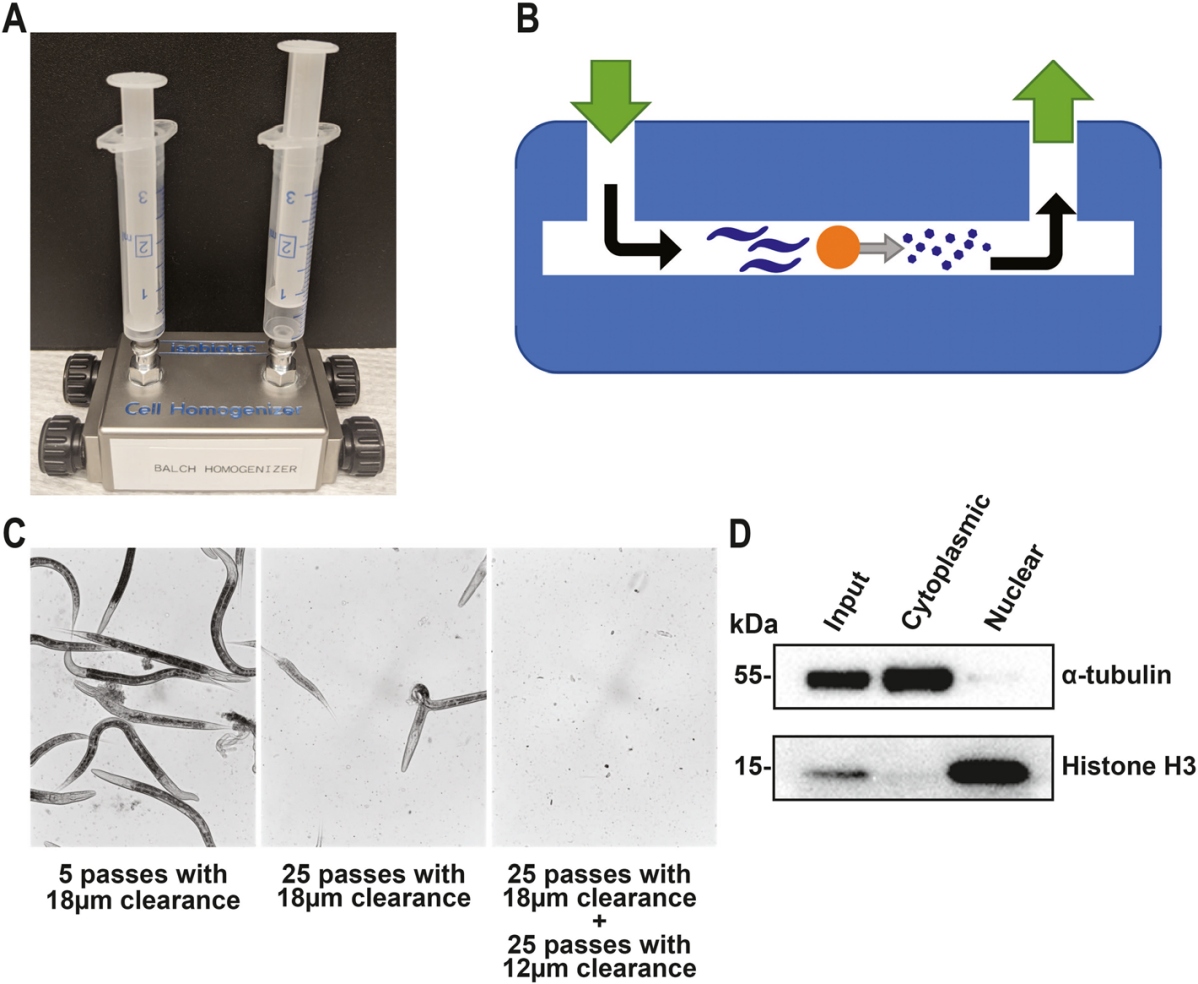
Preparation of *C. elegans* nuclear extract. **a** A photo of Balch homogenizer. **b** A schematic of Balch homogenizer. The chamber of Balch homogenizer was loaded with a ball bearing (the orange circle), and worms were suspended in complete hypotonic buffer. Worms were pushed through the gap between the chamber wall and the ball bearing. This movement was repeated to slowly break open the worms and produce a homogenate. **c** Worm lysates after different combinations of syringe pass numbers and ball-bearing sizes. **d** Western blot of fractionated homogenate. α-tubulin and histone H3 were probed as markers for the cytoplasmic fraction and nuclear extract, respectively. Input, 5 μg of homogenate before fractionation; cytoplasmic, 5 μg of the cytoplasmic fraction; nuclear, 5 μg of the nuclear extract

Although subcellular fractionation is widely used to prepare nuclear extract from cultured cells or animal tissues, subcellular fractionation of *C. elegans* is challenging because of the worms’ thick surrounding cuticle. Several techniques were described to break worms, including pressure cycling technology, bead beating, grinding after flash cooling, sonication, Dounce homogenization, and Balch homogenization [7–11]. The advantages and disadvantages of these methods have been reviewed in the literature [7–11]. For example, the pressure cycling technology can achieve complete disruption of worms through high pressure, but it requires expensive equipment and mixing of animals with an SiC abrasive. Bead beating is cheap and fast, but it results in highly variable protein recoveries as well as protein aggregation and denaturation. Grinding after flash cooling is rapid, but it needs a large amount of starting materials due to significant sample loss. Sonication and Dounce homogenization are effective but could lead to protein instability. In contrast, Balch homogenization, which uses a proprietary device Balch homogenizer (Fig. 1a), was shown by Bhaskaran et al. [7] to effectively disrupt worms and yield functional protein extracts. This method also generates amounts of soluble proteins that are comparable to sonication or Dounce homogenization, while also keeping proteins, nucleic acids, mitochondria, and polysomes intact [7]. After surveying the literature, we decided to test Balch homogenization for nuclear extraction in our *C. elegans* transcription system because of its reported advantages and the device’s affordable price (~$2000 each).

A Balch homogenizer consists of a hollow metal chamber into which a ball bearing of defined diameter is inserted (a schematic is depicted in Fig. 1b). The gap or clearance between the chamber wall and the ball bearing is adjustable by using ball bearings of different diameters. Worms in a buffer can be repeatedly pushed through the gap back and forth from the two syringes connected to the two ends of the chamber to generate homogenized lysate (homogenate). The homogenizer can also be placed on ice during operation to keep the sample from overheating. We tested different combinations of syringe pass numbers and ball-bearing sizes for optimal homogenization and observed that 25 passes with an 18 μm-clearance ball bearing followed by 25 passes with a 12 μm-clearance ball bearing resulted in complete disruption of L4 larvae or young adult worms (Fig. 1c). This procedure was then used for all worm homogenization in the studies described below. After homogenization, the cytoplasmic fraction was separated from the nuclei by centrifugation, and the nuclei were lysed in a hypotonic buffer to obtain nuclear extract. We subsequently checked the effectiveness of our subcellular fractionation through Western blot analyses of the cytoplasmic fraction and nuclear extract using α-tubulin and histone H3 as marker proteins, respectively [17]. Western results showed that α-tubulin mainly appeared in the cytoplasmic fraction and not in the nuclear fraction, whereas histone H3 was exclusively detected in the nuclear extract (Fig. 1d), indicating that proper subcellular fractionation was achieved. Because of this success, we did not test the usefulness of the other worm-breaking methods for nuclear extraction in our system.

### Detection of in vitro *C. elegans* transcription by PCR and gel eletrophoresis

**Fig. 2:**
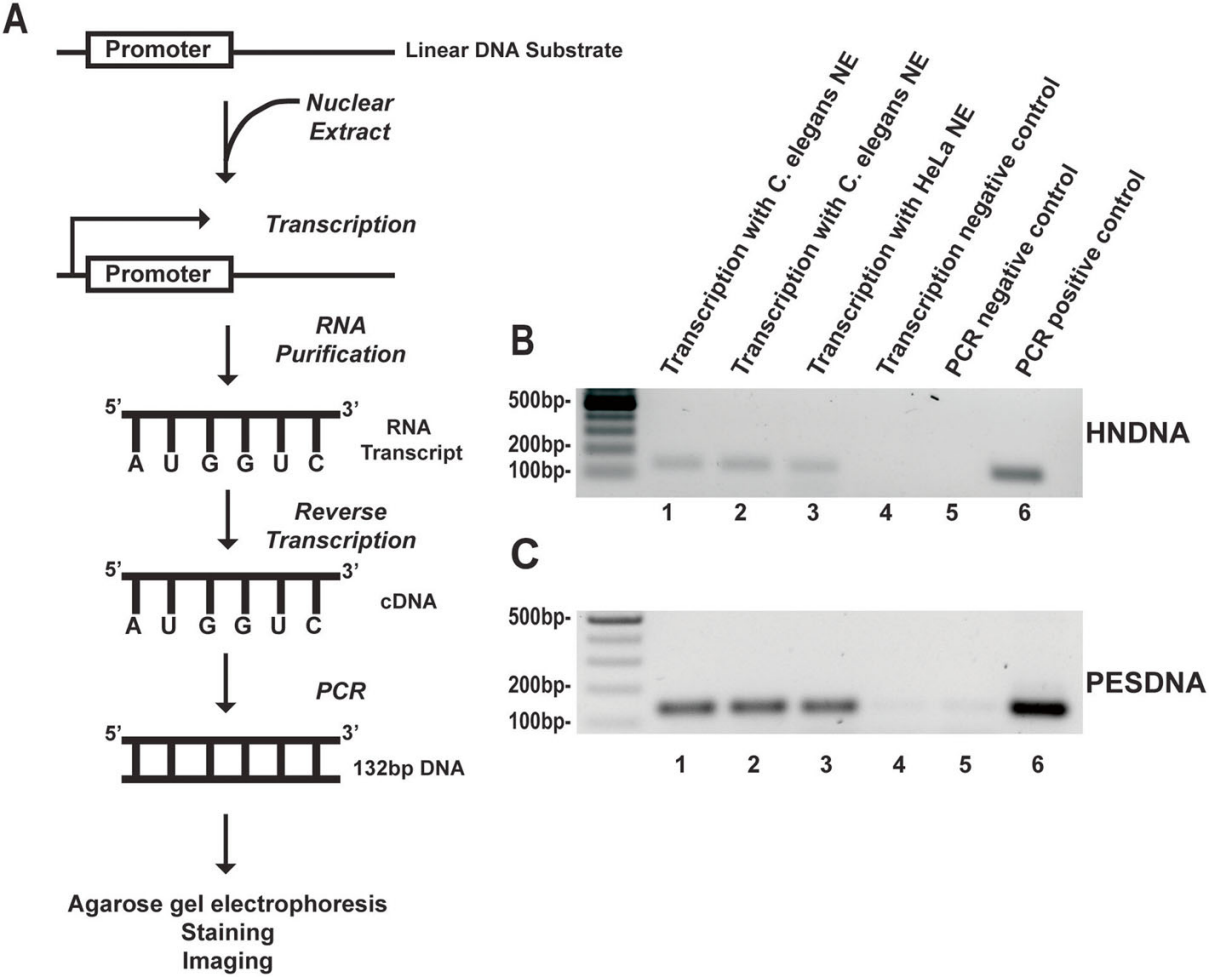
Detection of *C. elegans* transcription by PCR and gel electrophoresis. **a** Scheme of gel-based detection of *C. elegans* transcription. A linear DNA containing a transcription promoter was used as the template. Nuclear extract was added into the reaction to synthesize RNA, followed by RNA purification and reverse transcription. The resulting cDNA was amplified by PCR. The DNA product was then run on agarose gel, stained with SYBR Safe Stain, and imaged with a CCD camera. **b** and **c** Gel images of the products of transcription reactions with HNDNA **b** or with PESNDA **c**. Lanes 1 and 2, transcription with *C. elegans* nuclear extract; lane 3, transcription with HeLa nuclear extract; lane 4, transcription negative control (without any nuclear extract); lane 5, PCR negative control (without any nucleotides); lane 6, PCR positive control

After succeeding in preparation of *C. elegans* nuclear extract, we proceeded to re-constitute transcription reactions in vitro using such extract. Traditional in vitro transcription assays rely on radioactive labeling of the newly synthesized RNA and detection of incorporated radioactivity by autoradiography [13]. However, as technology has advanced, non-radioactive detection methods were developed. Voss et al. reported a simple qualitative PCR detection of RNA transcripts [15]. We decided to adapt this method to detect transcription of *C. elegans* nuclear extract. Figure 2a depicts the scheme of our experimental procedure. Briefly, a linear DNA containing the CMV promoter (HNDNA) was used as the template (DNA sequence is listed in Table S1). Nuclear extract was added to the DNA template to synthesize RNA, followed by RNA purification and reverse transcription. The resulting cDNA was amplified by PCR with the primer pair *HNqPCRrev1* and *HNqPCRfrw1* (Table S1) to produce a 132-bp DNA. The amplified DNA was then run on agarose gel, followed by staining and imaging. Because HeLa nuclear extract was proven to work efficiently in in vitro transcription assays [18], we first set up transcription reactions using the HeLa Scribe Nuclear Extract in vitro Transcription System. However, instead of using radioactive nucleotides and autoradiography detection, we followed the above-described PCR protocol. As shown in Fig. 2b, a DNA fragment of expected size (132 bp) was produced, confirming that the PCR method can amplify and detect the RNA transcript. Substitution of HeLa nuclear extract with *C. elegans* nuclear extract in the transcription reactions generated a DNA product of the same size (Fig. 2b), indicating that the *C. elegans* nuclear extract was capable of re-constituting transcription and that the nematode can use the CMV promoter to transcribe DNA. This notion was confirmed by in vivo experiments where we generated a transgenic worm strain by inserting the CMV promoter upstream of the *gfp* gene in plasmid pPD95.77 and injecting the resulting construct into wild-type *N2* worms. We observed GFP expression in the transgenic worms’ intestine, especially in the anterior and the rear regions, whereas worms injected with the control plasmid pPD95.77 lacking CMV showed no GFP expression (Fig. S1). These results demonstrate that, like in mammals, the CMV promoter indeed can drive gene expression in *C. elegans*.

*Δpes-10*, the minimal promoter of the worm gene *pes-10*, was shown to support strong gene expression in *C. elegans* [19, 20]. We constructed a *Δpes-10*-containing DNA substrate, PESDNA, that is structurally similar to the CMV-containing substrate HNDNA (Table S1). Combining PESDNA with *C. elegans* nuclear extract or HeLa nuclear extract in the transcription system yielded a DNA product with the expected size (132 bp) (Fig. 2c), indicating that *Δpes-10* can drive transcription in the in vitro system.

We next attempted to adapt this PCR method to quantitatively measure the transcription activity of *C. elegans* nuclear extract by quantifying DNA in gel images using the ImageJ software. Although the quantification results were consistent between replicates in the same experiments, large variations were observed between experiments (data not shown), which indicates that this PCR method is not a good quantitative approach, possibly due to the multiple steps involved in the process (i.e., conversion of RNA into cDNA, amplification of cDNA by PCR, gel electrophoresis, and densitometric measurement using ImageJ). Taken together, we have successfully adapted a PCR method to qualitatively detect in vitro *C. elegans* transcription.

### Quantification of in vitro *C. elegans* transcription activity by qRT-PCR

**Fig. 3:**
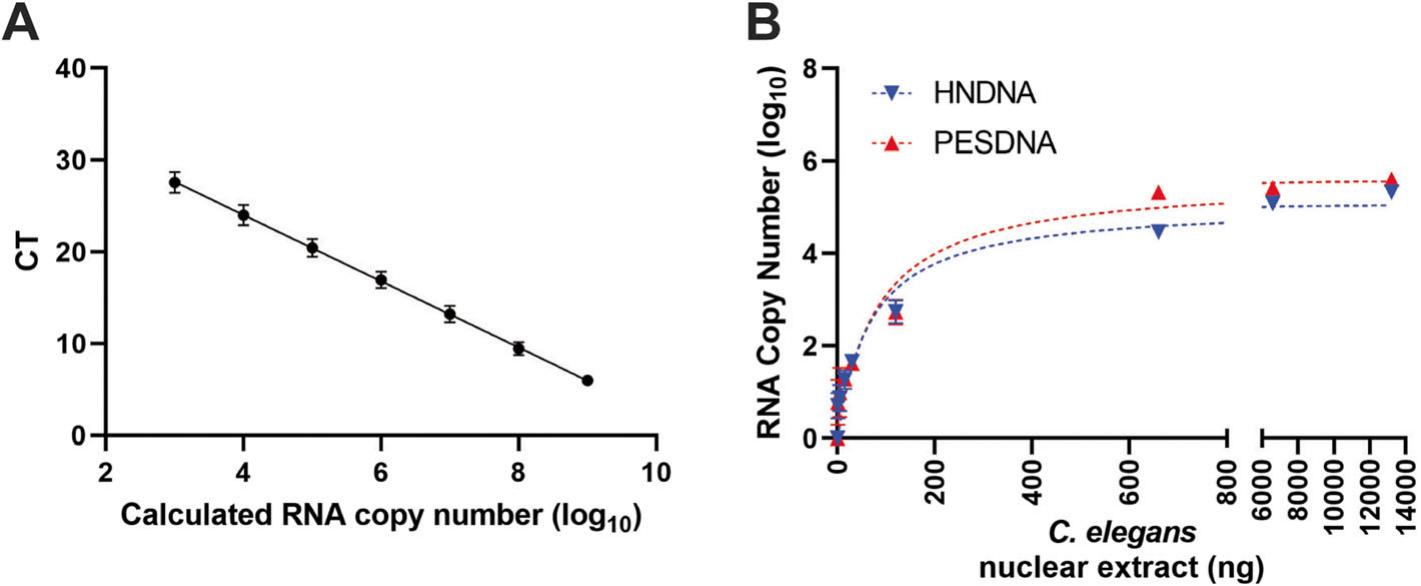
Quantitative analysis of RNA transcription by qRT-PCR. In vitro transcription was performed, and the resulting RNA was reverse transcribed into cDNA, followed by qRT-PCR quantification of the cDNA. **a** The template DNA was serially diluted 10-fold for seven times and subjected to qRT-PCR. The cycle thresholds (CTs) were plotted against the expected copy numbers of the template DNA, showing a linear relationship (Y = − 3.677x + 40.12, *r*^*2*^ = 0.9628). This standard curve was then used for the calculation of RNA copy number generated from transcription reactions. The graph represents the combined results of three independent experiments. Error bars represent standard deviation. **b** PESDNA or HNDNA was titrated with *C. elegans* nuclear extract in the in vitro transcription system. The RNA copy numbers generated from transcription were plotted against the amounts of *C. elegans* nuclear extract used in the reactions, followed by fitting with the Michaelis-Menten model and the non-linear least-squares method in Excel spreadsheets. The graph represents the combined results of three independent experiments. Error bars represent standard deviation

We next sought to quantify *C. elegans* transcription activity using qRT-PCR, a more direct approach than the above-described gel-based method because it amplifies DNA and simultaneously measures DNA amounts using a fluorescent reporter. To this end, we followed the same experimental procedure as depicted in Fig. 2a to generate cDNA and then amplified cDNA by qRT-PCR with SYBR green detection. To assess the quantifiability of this method, we first conducted transcription reactions using HNDNA and various amounts of *C. elegans* nuclear extract followed by qRT-PCR. Because in qRT-PCR the intensity of fluorescent signal is proportional to the number of amplified DNA molecules [21], a standard curve of the template DNA was included in the assays and used for RNA copy number calculations under the assumption that every RNA molecule was reverse-transcribed into a DNA molecule (Fig. 3a). Conversion of fluorescent signals to RNA copy numbers and fitting of the latter with the Michaelis-Menten model and the non-linear least-squares method, as described previously [22], yielded a coefficient of determination (*r*^*2*^) of 0.9491 (Fig. 3b), indicating that the enzymatic reactions in transcription follows the Michaelis-Menten model and that the qRT-PCR method allows for quantitative measurement of transcription activity. We next titrated PESDNA with various amounts of *C. elegans* nuclear extract, followed by conversion of fluorescent signals to RNA copy numbers and data fitting (Fig. 3b). Two titration parameters, the maximum yield and the amount of nuclear extract needed to reach 50% of the maximum yield, are listed in Table 1. Compared to the titrations with HNDNA, the titrations with PESDNA yielded 3-fold maximum RNA molecules and needed 1.37-fold nuclear extract to reach 50% of the maximum yield (Table 1), indicating that the transcription reactions with PESDNA were more robust and that PESDNA was a better substrate than HNDNA in the reactions.

**Table 1:** Parameters of transcription titrations with *C. elegans* nuclear extract

DNA substrate	Maximum RNA copy number	Amount of nuclear extract needed to reach 50% of the maximum yield (ng)
HNDNA	117,220	68.44
PESDNA	354,813	93.66

### Inhibition of in vitro *C. elegans* transcription

**Fig. 4:**
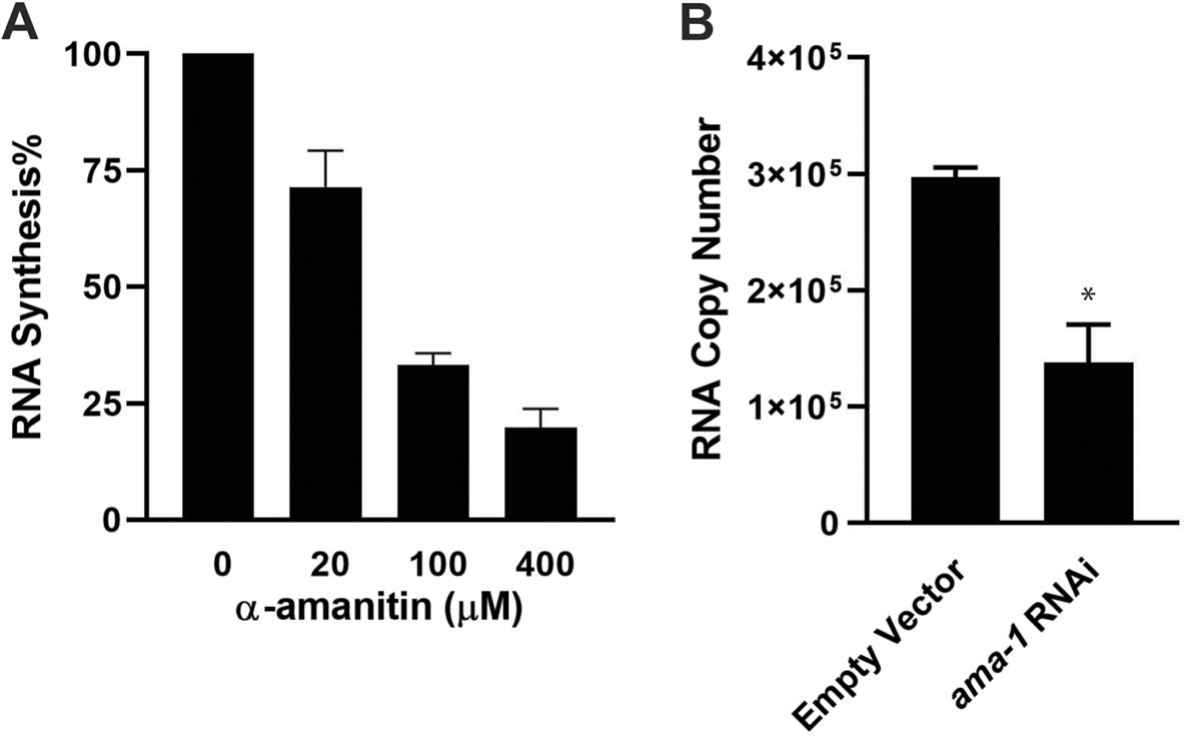
Inhibition of in vitro *C. elgeans* transcription. **a** α-amanitin was added into transcription reactions containing *C. elegans* nuclear extract and PESDNA, followed by qRT-PCR quantification. The RNA copy numbers were normalized against reactions with 0 μM α-amanitin. The graph represents the combined results of three independent experiments. **b** Nuclear extract prepared from *ama-1* RNAi wroms or control worms was used for transcription reactions, followed by qRT-PCR quantification. The graph represents the combined results of three independent experiments. Asterisk (*) denotes a significant difference (*P* < 0.05) between *ama-1* RNAi and the control, as analyzed by a two-sample *t* test for independent samples

To test the responsiveness of our in vitro system to transcription inhibition, we subjected the system to two types of inhibition: addition of the RNA Pol II inhibitor α-amanitin in transcription reactions and RNAi of *ama-1*, the gene that encodes the RNA Pol II subunit A, in *C. elegans* followed by nuclear extraction and transcription assays. α-amanitin is a well-known toxin that directly inhibits RNA pol II [23]. We added various amounts of α-amanitin (0, 20, 100, or 400 μM) into transcription reactions that contain *C. elegans* nuclear extract and the PESDNA substrate, followed by qRT-PCR quantification. Results showed that production of RNA molecules decreased as the concentration of α-amanitin increased in the reactions (Fig. 4a). This demonstrates that the in vitro system is responsive to α-amanitin inhibition and indicates that the in vitro transcription is indeed carried out by RNA pol II.

The gene *ama-1* encodes the RNA Pol II subunit A of *C. elegans*, and inactivation of *ama-1* by RNAi blocks embryonic transcription and arrests embryo development [24]. We performed RNAi of *ama-1* by feeding L2 worms *E. coli* strain HT115(DE3) with a vector expressing double-stranded RNA that is homologous to *ama-1* or with an empty vector as a negative control. After 48-h incubation, *C. elegans* nuclear extract was isolated, followed by in vitro transcription and qRT-PCR quantification. Results showed that the nuclear extract isolated from *ama-1*-silenced worms had a lower transcription activity than that from control worms (Fig. 4b). Taken together, both α-amanitin and RNAi of *ama-1* suppressed the transcriptional activity in our system, consistent with the inhibitory effects of these treatments on RNA Pol II. This illustrates the responsiveness of our system to transcription inhibition and indicates the usefulness of our system in evaluating transcription activity under different conditions.

### Comparison of *C. elegans* transcription activity with or without *P. aeruginosa* infection

**Fig. 5:**
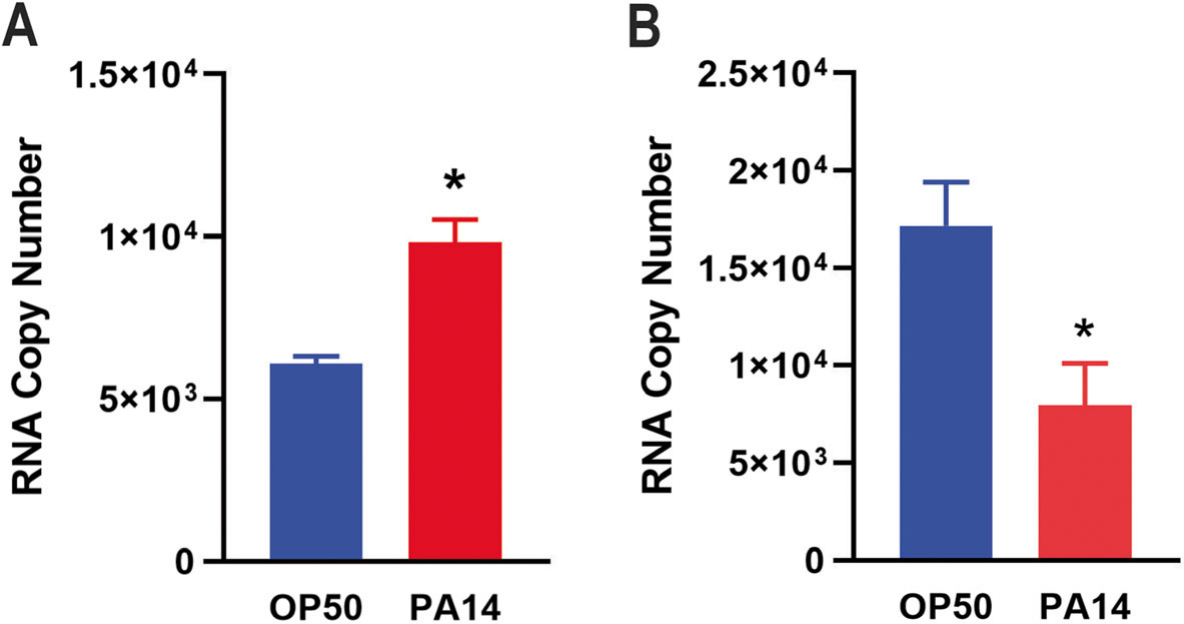
Comparison of *C. elegans* transcription activity with or without *P. aeruginosa* infection. Synchronized young-adult worms were exposed to *P. aeruginosa* PA14 or *E. coli* OP50 as a control. The worms were then collected and subjected to nuclear extraction, followed by transcription assays with either HNDNA **a** or PESDNA **b**. The graphs represent the combined results of three independent experiments. Error bars represent standard deviation. Asterisk (*) denotes a significant difference (*P* < 0.05) between infected worms and uninfected control worms, as analyzed by a two-sample *t* test for independent samples

Next we applied the above-described transcription system to assess how pathogen infection affects *C. elegans* transcription activity. Upon infection, host organisms launch stress responses to fight invading microbes, in part, by altering gene expression [25]. Our in vitro *C. elegans* transcription system offers a suitable approach to examine how RNA pol II changes transcription activity at the whole-organism level. To this end, we chose the human opportunistic pathogen *P. aeruginosa* strain PA14 for worm infection, because the *P. aeruginosa*-*C. elegans* infection model is well-established for bacterial pathogenesis research [26–28]. To infect worms, young-adult worms fed on standard worm food *Escherichia coli* strain OP50 were transferred to a lawn of *P. aeruginosa* and incubated at 25 °C for 1 h. Uninfected control worms stayed on *E. coli* at 25 °C for 1 h. These worms were then collected, nuclear extract was prepared using Balch homogenization, and transcription assays were conducted with qRT-PCR quantification, as described above. Results showed that when HNDNA was used as the substrate, on average, 1 μg of nuclear extract from uninfected worms transcribed 6072 RNA molecules, while infected nuclear extract transcribed 9837 RNA molecules, a 1.62-fold increase in transcription activity upon infection (Fig. 5a). By contrast, when PESDNA was used as the substrate, 1 μg of nuclear extract from uninfected worms transcribed 17,162 RNA molecules, while infected nuclear extract transcribed 7959 RNA molecules, a 2.16-fold decrease upon infection (Fig. 5b). These results revealed that even in the in vitro system, transcription activity is promoter- or gene-specific, supporting that our system can be useful for studying gene expression regulation.

## Discussion

Even though *C. elegans* has been used as a model system to address a wide range of biological questions, the nematode’s contributions to biochemical elucidation of eukaryotic transcription mechanisms have been limited. One of the biggest hurdles in in vitro studies of *C. elegans* transcription is the high difficulty of obtaining functionally active nuclear extract due to the nematode’s thick surrounding cuticle. By adapting the method of Balch homogenization [7], we achieved effective worm disruption and optimal subcellular fractionation and obtained nuclear exact that was functionally active in inducing transcription appropriately. A non-radioactive, PCR-based detection method was also adapted into our transcription system. The PCR method can qualitatively detect *C. elegans* transcription in vitro by gel electrophoresis or quantitatively measure transcription activity through qRT-PCR. Applying this system to assess how pathogen infection affects *C. elegans* transcription revealed that *P. aeruginosa* infection can change transcription activity and the change is promoter- or gene-specific. Therefore, our in vitro system can be a useful tool for biochemically studying transcription mechanisms and gene expression regulation in *C. elegans*, which could facilitate the understanding of transcription in higher organisms due to the conservation of eukaryotic transcription.

As in other eukaryotes, the RNA Pol II-mediated transcription in *C. elegans* depends on the binding of transcription factors to specific gene *cis*-acting sequences [3]. These *cis*-regulatory sequences are usually clustered into discrete functional modules including the core promoter, extended proximal and downstream promoter regions, positive and negative enhancers, and insulators. Pol II acts in concert with TATA Binding Protein (TBP) and TBP-Associated Factors (TAFs) at the core promoter to initiate transcription. The core promoter in *C. elegans* typically includes five elements: an Sp1 like site (CNCCGCCC), T-blocks that correlate with nucleosome eviction and gene expression levels (TTTT [N/T]), a TATA box (GTATA [TA][TA]AG), a trans-splicing site (TTnCAG), and a Kozak site that includes the translation initiation codon ([CA] AA [CA]ATG) [29]. One of the DNA substrates used in our in vitro system is HNDNA, which contains the CMV promoter and other minimal elements required to induce transcription using nuclear extract from mammalian cells [15]. Its core promoter contains all five elements (or similar sequences) typical for *C. elegans* and was able to induce transcription appropriately with nematode nuclear extract. These results suggest that, like in mammals, the CMV promoter can drive gene expression in *C. elegans*. Mao et al. reported that the Tetracycline (Tet)-controlled transcription system, which contains the CMV minimal promoter, is largely inactive in *C. elegans* [20]. This could be due to the competitive binding of unknown factors in *C. elegans* to the Tet operator sequences upstream the CMV promoter, which would interfere with the binding of the activator rTetR-VP16 to the same sequences. Such competition for binding could also explain that little transcription was observed even when the CMV promoter was replaced with the *Δpes-10* promoter, whereas substitution of rTetR-VP16 with rTetR-QFAD induced strong transcription [20]. Our in vitro system will be useful for biochemically studying *C. elegans* transcription machinery components. For example, because a custom DNA template is used in this system, the role of any *cis*-acting sequence can be investigated by including or excluding the specific sequence in the template. Similarly, transcription factor functions can be studied by depleting or complementing the nuclear extract with specific factors. This system should be especially suitable for examining the interactions of transcription factors and *cis*-acting sequences because the interacting complexes can be pulled down by using either specific antibodies against the proteins or streptavidin beads against a biotinylated DNA template. Many mechanistic questions regarding *C. elegans* transcription that could not be answered previously can now be biochemically interrogated using our system. However, one drawback of this system is that spatial regulation of transcription, such as cell- or tissue-specific gene expression, cannot be investigated because the nuclear extract is prepared from whole animals.

The usefulness of our transcription system in solving biological problems was demonstrated by the comparison of *C. elegans* transcription activity with or without *P. aeruginosa* infection, which revealed that infection can change the nematode’s transcription activity and the change is promoter- or gene-specific. These results suggest that our system can be useful for studying gene expression regulation such as transcriptional responses to environmental or internal assaults. Therefore, our in vitro transcription system not only fills a technical gap in biochemical studies of *C. elegans*, but also expand the usefulness of this powerful model organism in addressing many biological questions.

## Conclusions

In this study, we developed an in vitro *C. elegans* transcription system that re-constitutes transcription reactions using nuclear extract of larval or adult worms, and can both qualitatively and quantitatively detect transcription activity using a non-radioactive approach. This in vitro system employs Balch homogenization to effectively disrupt worms followed by subcellular fractionation, resulting in a nuclear extract that is functionally active in inducing transcription appropriately. We adapted a non-radioactive, PCR-based detection method to either qualitatively or quantitatively detect in vitro *C. elegans* transcription. Overall, this system will be useful for biochemically studying *C. elegans* transcription mechanisms and gene expression regulation. More specifically, it can be used to study *C. elegans* transcription machinery components, such as transcription factors, *cis*-acting sequences, or interactions between them; it can also be used to examine gene expression regulation under specific environmental or internal conditions. Knowledge gained from these studies will also facilitate our understanding of transcription in higher organisms due to the conservation of eukaryotic transcription. Furthermore, the ability to effectively prepare functionally active nuclear extract for use in our in vitro system fills a technical gap in biochemical studies of *C. elegans* and will expand the usefulness of this powerful model organism in addressing many biological questions beyond transcription.

## Methods

### Bacterial and *C. elegans* strains

*E. coli* OP50 and *P. aeruginosa* PA14 were used in this study. These bacteria were grown in Luria-Bertani (LB) broth at 37 °C. Wild-type *C. elegans* Bristol N2 was used and cultured under standard conditions [30].

### Worm disruption and subcellular fractionation

Gravid adult animals were lysed using a solution of sodium hydroxide and bleach (ratio 5:2), and eggs were synchronized for 22 h in S-basal liquid medium at room temperature. Synchronized L1 larval animals were transferred onto NGM plates seeded with *E. coli* OP50 and grown at 20 °C for 48 h until the animals reached L4 larval stage or for 72 h until young adult stage. The animals were then collected, washed with M9 buffer, and used for nuclear extract preparation directly or for pathogen infection followed by nuclear extract preparation. For pathogen infection, the collected worms were transferred to NGM plates containing *E. coli* OP50 or *P. aeruginosa* PA14 at 25 °C and incubated for 1 h. Following this incubation, the animals were collected and washed with M9 buffer three times. The animals were then washed again in 3 ml cold hypotonic buffer (15 mM HEPES KOH, pH 7.6, 10 mM KCl, 5 mM MgCl_2_, 0.1 mM EDTA, and 350 mM Sucrose) and centrifuged at 1425 x g for 3 min at room temperature. The supernatant was discarded, and the animal pellet was resuspended in 1 ml of cold hypotonic buffer with 2x Protease inhibitor (ThermoFisher, catalog # 78430). The animals were transferred to a Balch homogenizer and grounded using an 18 μm clearance ball bearing for 25 passes, followed by another 25 passes using a 12 μm ball bearing. The final animal homogenate was transferred to a 1.5 ml microtube and centrifuged at 500 x g for 5 min at 4 °C. The supernatant was then transferred to a new 1.5 ml microtube, and 40 μl of supernatant was aliquoted to a microtube labeled “input”. The remaining supernatant was centrifuged at 4000 x g for 5 min at 4 °C to pellet the nuclei. The supernatant was transferred to a new microtube and centrifuged at 17,000 x g for 30 min at 4 °C, and the supernatant was collected as the “cytoplasmic fraction”. The nuclei pellet was washed with 500 μl of complete hypotonic buffer and centrifuged at 4000 x g for 5 min at 4 °C. The supernatant was discarded, and the nuclei pellet was resuspended in 500 μl of complete hypotonic buffer and transferred to a new microtube. The resuspended nuclei was then centrifuged at 4000 x g for 5 min at 4 °C. After discarding the supernatant, the nuclei pellet was resuspended in 40 μl cold hypertonic buffer (15 mM HEPES KOH, pH 7.6, 400 mM KCl, 5 mM MgCl_2_, 0.1 mM EDTA, 0.1% Tween20, 10% Glycerol, 2x Protease inhibitor) and the suspension was transferred to a new microtube as the “nuclear fraction”. Fractions were quantified using Invitrogen Qubit protein assay (ThermoFisher, catalog # Q33221) before snap freezing. All samples were stored at − 80 °C until use in the in vitro transcriptional assay.

### Western blot

Western blotting was performed as previously described [31]. Briefly, equal mass aliquots of isolated cytoplasmic, nuclear, and input fractions were diluted with H_2_O to 7.5 μl, mixed with 2.5 μl of NuPAGE LDS buffer (Invitrogen, catalog # NP0007), and heated to 70 °C for 10 min for denaturation. The isolates were separated on a NuPAGE 4–12% Bis-tris gel and transferred to a PVDF membrane. The primary antibodies used were mouse anti-tubulin alpha antibody (AA43) developed by Walsh, C (obtained from the Developmental Studies Hybridoma Bank at the University of Iowa, Department of Biology) and rabbit anti-histone H3 antibody (Novus Biologicals, catalog # NB500–171). The secondary antibodies used were goat anti-mouse IgG (H + L) antibody conjugated to HRP (Invitrogen, catalog # 31430) and goat anti-rabbit IgG (H + L) antibody conjugated to HRP (Promega, catalog # W4018). Immunoblots were imaged using iBright 1500 (ThermoFisher, catalog # A44241).

### In vitro transcription assay and RNA purification

Transcription assays were set up based on the HeLa Scribe Nuclear Extract in vitro Transcription system (Promega, catalog # E3110) with modifications. A master mix was prepared to include 1.5 μl of 50 mM MgCl_2_, 1.0 μl of rNTPs (10 mM of each), 4 μl of linear HNDNA or PESDNA (25 ng/μl), and 7.5 μl of RNase-free H_2_O. Assay tubes were filled with 5 μg of nuclear extract and transcription buffer to a total volume of 11 μl. Fourteen μl of master mix was transferred to each assay tube, and the reactions were incubated at 30 °C for 30 min. Two control reactions were performed: one containing 8 units of HeLa nuclear extract provided by Promega as a positive control and the other containing no nuclear protein as a negative control. Reactions were halted with the addition of 400 μl of RLT buffer from the RNeasy Micro kit (Qiagen, catalog # 74004), snap frozen, and stored at − 80 °C until RNA cleanup. RNA cleanup was done using the RNeasy Micro kit following the manufacture’s recommendations, including on-column DNase treatment but excluding the addition of carrier RNA. The purified RNA was eluted from the column using 17 μl of RNase-free H_2_O. Eluted RNA was again treated with DNase using the Baseline-ZERO DNase kit (Lucigen, catalog # DB0715K) following the manufacturer’s instructions. All RNA samples were stored at − 80 °C until reverse transcription.

### PCR amplification of transcription products

Reverse transcription reactions containing 2 μl of purified RNA and 2 μl of 10 mM *HNqPCRrev1* primer in a final volume of 20 μl were performed using the Qiagen Sensiscript RT kit (Qiagen, catalog # 205211) per the manufacturer’s instructions. After the reaction was completed, 1 μl of the reverse transcription mix was transferred to a PCR tube and used for PCR. The standard PCR reaction was performed with the *HNqPCRrev1* and *HNqPCRfrw1* primers using the Failsafe PCR system with the Premix A (Lucigen, catalog # F599100). Positive and negative PCR control reactions with or without 50 ng of HNDNA or PESDNA, respectively, were also performed. After amplification, the PCR products were analyzed by gel electrophoresis on a 2.0% agarose TAE gel, followed by staining with SYBR safe DNA gel stain (Invitrogen, catalog # S33102), imaging with iBright 1500, and quantification with software ImageJ.

### qRT-PCR

Quantification by qRT-PCR was done on the StepOnePlus 96-well real-time PCR system (Applied Biosystems, catalog # 4376600) using the PowerUp SYBR green qPCR kit (Applied Biosystems, catalog # A25918). Two microliters of reverse transcription product were amplified using 500 nM each of the *HNqPCRrev1* and *HNqPCRfrw1* primers in a 10 μl reaction. A seven-point standard curve of the linear HNDNA or PESDNA was included in every qRT-PCR experiment and used for RNA copy number calculations under the assumption that every RNA molecule was reverse-transcribed into a DNA molecule. Titrations of *C. elegans* nuclear extract were performed using the in vitro transcription assay followed by qRT-PCR quantification. The DNA products were converted to RNA copy numbers using the standard curve of the linear DNA and then fitted with the Michaelis-Menten model and the non-linear least-squares method in Excel spreadsheets, as described previously [22]. The resulting fitting equation was used for calculating the maximum yield of RNA copy number and the amount of nuclear extract needed to reach 50% of the maximum yield.

### *ama-1* RNA interference

Gravid adult worms were lysed using a solution of sodium hydroxide and bleach (ratio 5:2), and eggs were synchronized for 22 h in S-basal liquid medium at room temperature. Synchronized L1 larval worms were transferred onto NGM plates seeded with *E. coli* OP50 and grown at 20 °C for 24 h until the worms passed L2 larval stage. The animals were then collected, washed with M9 buffer, transferred to NGM plates containing *E. coli* HT115(DE3) expressing double-stranded RNA (dsRNA) that is homologous to *ama-1* or *E. coli* HT115(DE3) with an empty vector. The worms were incubated at 20 °C for 48 h and then used for in vitro transcription assays. *Unc-22* RNAi was included as a positive control in all experiments to account for RNAi efficiency.

### Plasmid construction and transgenic worm generation

The CMV promoter (507 bp) was spliced from HNDNA via *SpeI* and *XbaI* and cloned into plasmid pPD95.77 digested with *XbaI*. This resulted in plasmid pPW04. Transgenic strain JRS78 was generated by injecting pPW04 (150 ng/μl with a *rol-6(su1006)* co-injection marker at 10 ng/μl) into Bristol *N2* wild-type worms. Control worms (strain JRS79) were generated by injecting the pPD95.77 backbone lacking the CMV promoter at 150 ng/μl with a *rol-6(su1006)* co-injection marker at 10 ng/μl.

### Fluorescence imaging

Before imaging, synchronized JRS78 or JRS79 worms were placed on empty NGM plates and allowed to crawl for 10 min to clean off any external bacteria. The worms were then immobilized using 30 mM sodium azide, transferred to freshly prepared 2% agarose pads, and covered with a 1-mm cover glass. Worms were imaged using a Zeiss Axio Imager M2 fluorescence stereomicroscope equipped with DIC and Zen 2 capture software.

## Supplementary Information

**Additional file 1: Table S1**. Sequences of template DNA and primers for PCR and qRT-PCR

**Additional file 2: Figure S1. GFP expression driven by the CMV promoter in** ***C. elegans*.** Transgenic worms were generated by inserting the CMV promoter upstream of the *gfp* gene in plasmid pPD95.77 and injecting the resulting construct into wild-type *N2* worms. Control worms were generated by injecting plasmid pPD95.77 lacking CMV. Worms were imaged using a Zeiss Axio Imager M2 fluorescence stereomicroscope equipped with DIC and Zen 2 capture software. DIC, differential interference contrast microscopy; GFP, GFP fluorescence microscopy; Merge, overlay of DIC and GFP images.

## Data Availability

All data generated or analyzed during this study are included in this published article.

## Abbreviations

PCR: Polymerase chain reaction
qRT-PCR: Quantitative reverse transcription polymerase chain reaction
NGM plates: Nematode growth media plates
CMV: Cytomegalovirus
HNDNA: HeLa Nuclear DNA Template
PESDNA: *Δpes-10* Promoter-Containing DNA Template
GFP: Green Fluorescent Protein
