# Supplementary material for: A novel in vitro Caenorhabditis elegans transcription system
Source: BMC Mol Cell Biol. 2020 Nov 30;21:87. doi: 10.1186/s12860-020-00332-8 (PMC7706227; doi:10.1186/s12860-020-00332-8)
Supplement: Supplementary file 1 — Additional file 1: Table S1. Sequences of template DNA and primers for PCR and qRT-PCR [file 12860_2020_332_MOESM1_ESM.docx]

| Table S1. Sequences of template DNA and primers for PCR and qRT-PCR | |
| --- | --- |
| Template or primer | Sequence 5’ → 3’* |
| HNDNA Template | ctcatgtttgacagcttatcgatccgggcaacgttgttgccattgtcgcaggcggagaactggtaggtatggaaggatctatacattgaatcaatattggccattagccatattattcattggttatatatcataaatcaatattggctattggccattgcatacgttgtatccatatcataatatgtacatttatattggctcatgtccaacattaccgccatgttgacattgattattgactagttattaatagtaatcaattacggggtcattagttcatagcccatatatggagttccgcgttacataacttacggtaaatggcccgcctggctgaccgcccaacgacccccgcccattgacgtcaataatgacgtatgttcccatagtaacgccaatagggactttccattgacgtcaatgggtggagtatttacggtaaactgcccacttggcagtacatcaagtgtatcatatgccaagtacgccccctattgacgtcaatgacggtaaatggcccgcctggcattatgcccagtacatgaccttatgggactttcctacttggcagtacatctacgtattagtcatccctattaccatggtgatgcggttttggcagtacatcaatgggcgtggatagcggtttcactcacggggatttccaagtctccaccccattgacgtcaatgggagtttgttttggcaccaaaatcaacgggactttccaaaatgtcgtaacaactccgccccattgacgcaaatgggcggtaggcgtgtacggtgggaggtctatatagcagagctcgtttagtgaaccgtcagatctctagaagctttaatgcggtagtttatcacagttaaattgctaacgcagtcaggcaccgtgtatgaaatctaacaatgcgctcatcgtcatcctcggcaccgtcaccctggatgctctaggcataggcttggttatgccggtactgccgggcctcttgcgggatatcgtccattccgacagcatcgccagtcactatggcgtgctgctagcgctatatgcgttgatgcaatttctatgcgcacccgttctcggagcactgtccgaccgctttggccgccgcccagtcctgctcgcttcgctacttggagccactatcgactacgcgatcatggcgaccacacccgtcctgt |
| PESDNA Template | ctcatgtttgacagcttatcgatccgggcaacgttgttgccattgtcgcaggcggagaactggtaggtatggaaggatctatacattgaatcaatattggccattagccatattattcattggttatatatcataaatcaatattggctattggccattgcatacgttgtatccatatcataatatgtacatttatattggctcatgtccaacattaccgccatgttgacattgattattgactagttattaatagtaatcaattacggggtcattagttcatagcccatatatggagttccgttgatatccctgcaggatcgattttttgcaaattacgagcgttgtagggggcggagcgataggtcctataggttttggtatatcatcattcattcattcattggtacattcatttacccaccttcctctttctgagcttctctggagttctgtgcttcctttttcccttatctttatactgtaatttttaactttcagggcgcgcccgtttagtgaaccgtcagatctctagaagctttaatgcggtagtttatcacagttaaattgctaacgcagtcaggcaccgtgtatgaaatctaacaatgcgctcatcgtcatcctcggcaccgtcaccctggatgctctaggcataggcttggttatgccggtactgccgggcctcttgcgggatatcgtccattccgacagcatcgccagtcactatggcgtgctgctagcgctatatgcgttgatgcaatttctatgcgcacccgttctcggagcactgtccgaccgctttggccgccgcccagtcctgctcgcttcgctacttggagccactatcgactacgcgatcatggcgaccacacccgtcctgt |
| Primer  *(HNqPCRfrw1)* | gccgggcctcttgcgggatat |
| Primer  *(HNqPCRrev1)* | cggccaaagcggtcggacagt |
| *Sequences highlighted in yellow are primer binding sites in the template DNA. | |
