# Supplementary figures and images for: A novel in vitro Caenorhabditis elegans transcription system
Source: BMC Mol Cell Biol. 2020 Nov 30;21:87. doi: 10.1186/s12860-020-00332-8 (PMC7706227; doi:10.1186/s12860-020-00332-8)

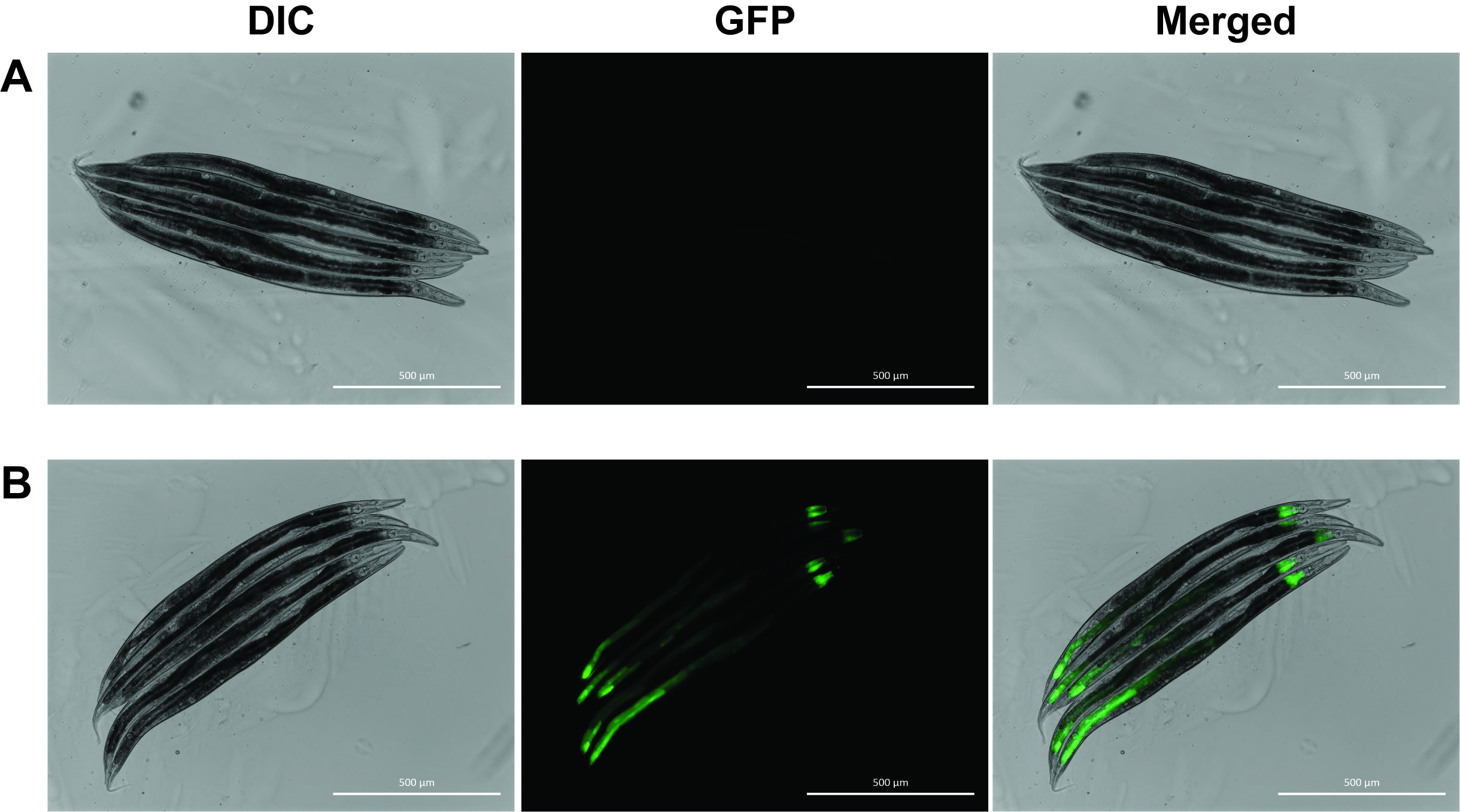

Supplement: Supplementary file 2 — Additional file 2: Figure S1. GFP expression driven by the CMV promoter in C. elegans. Transgenic worms were generated by inserting the CMV promoter upstream of the gfp gene in plasmid pPD95.77 and injecting the resulting construct into wild-type N2 worms. Control worms were generated by injecting plasmid pPD95.77 lacking CMV. Worms were imaged using a Zeiss Axio Imager M2 fluorescence stereomicroscope equipped with DIC and Zen 2 capture software. DIC, differential interference contrast microscopy; GFP, GFP fluorescence microscopy; Merge, overlay of DIC and GFP images. [file 12860_2020_332_MOESM2_ESM.jpg]
